# Supplementary material for: Different Effects of Albumin and Hydroxyethyl Starch on Low Molecular-Weight Solute Permeation through Sodium Hyaluronic Acid Solution
Source: Polymers (Basel). 2021 Feb 9;13(4):514. doi: 10.3390/polym13040514 (PMC7914834; doi:10.3390/polym13040514)
Supplement: Supplementary file 1 [file polymers-13-00514-s001.pdf]

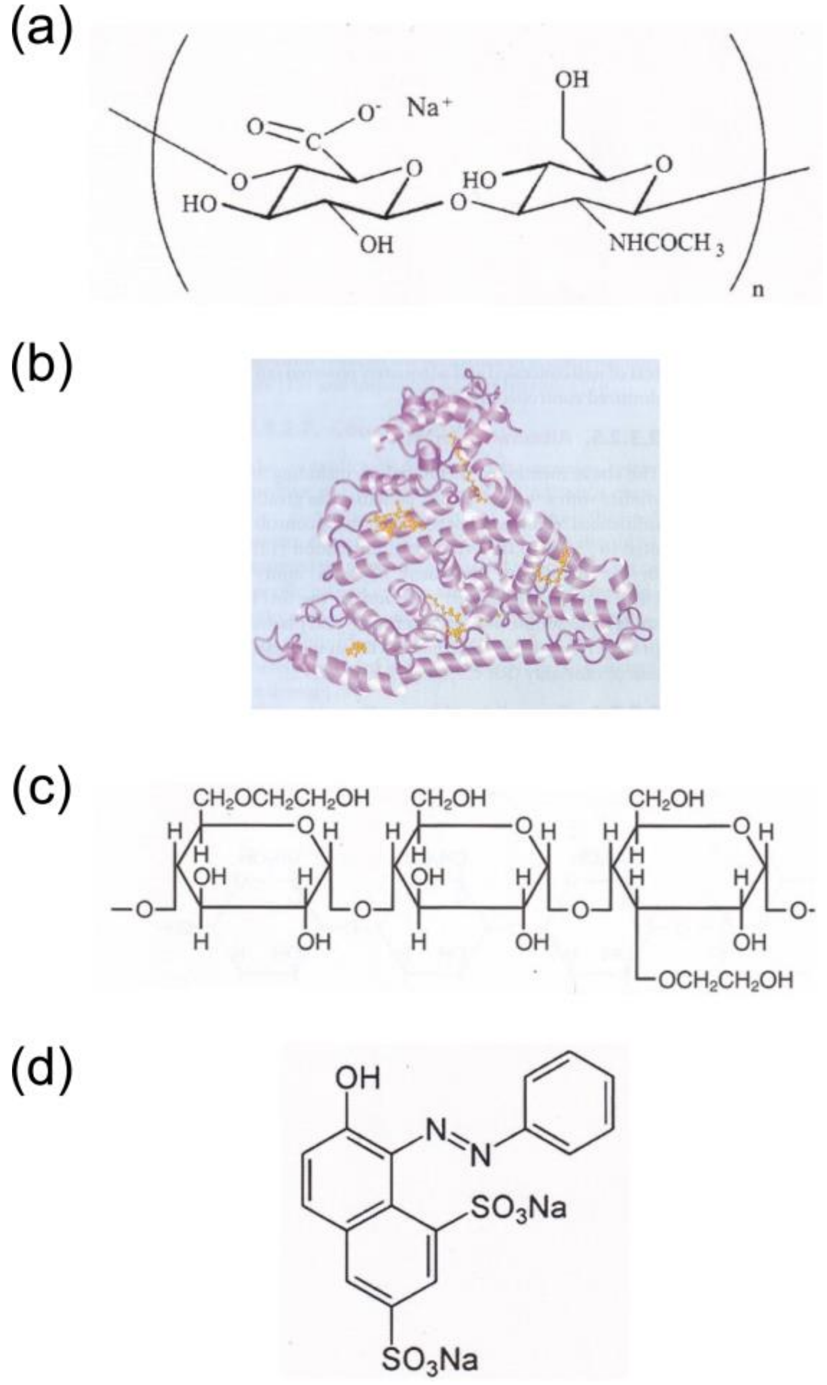


**Figure S1.** Chemical structures of (a) sodium hyaluronic acid, (b) albumin, (c) hydroxyethyl starch, and (d) Orange G.

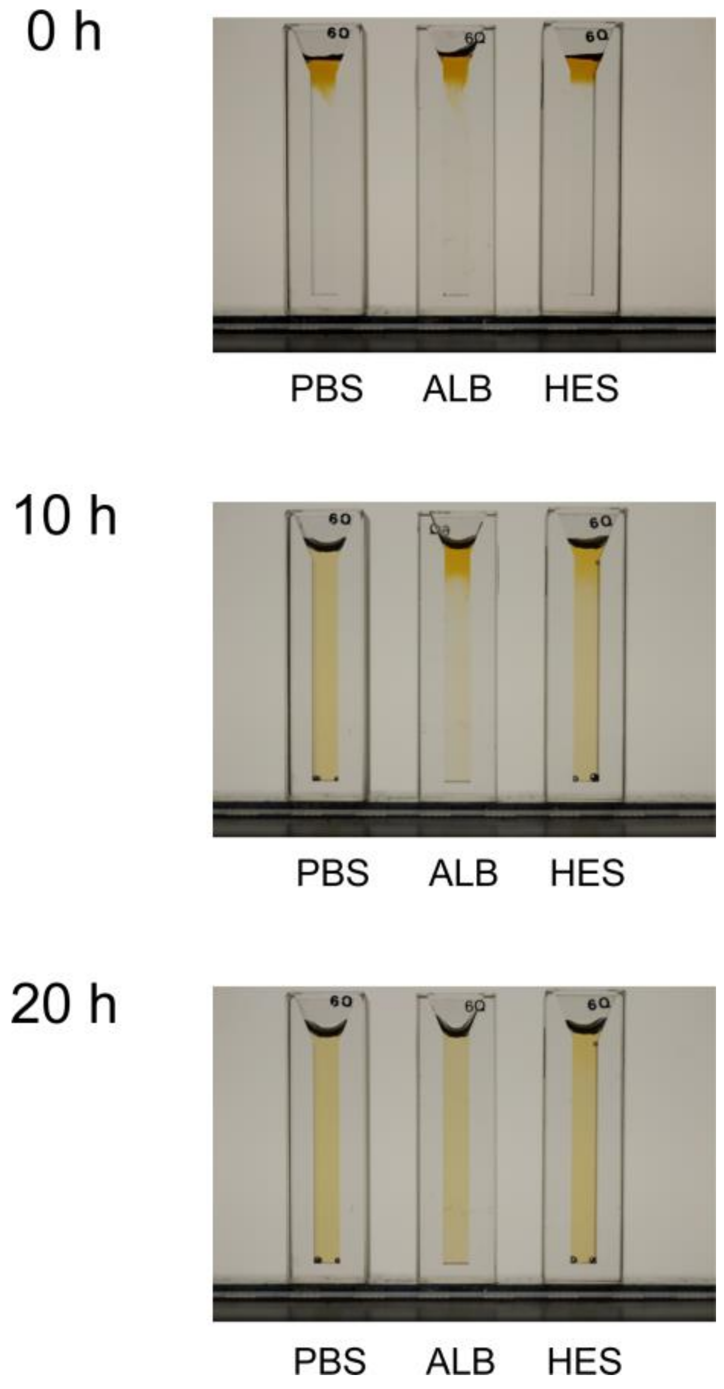


**Figure S2.** Direct visualization of Orange G permeation into 0.2% sodium hyaluronic acid in phosphate buffered saline (PBS), 1% albumin solution (ALB), and hydroxyethyl starch solution (HES) in ultraviolet cuvette at 37°C.
